# Chemical Composition and Antioxidant Activity of Prokupac Grape Pomace Extract: Implications for Redox Modulation in Honey Bee Cells

**DOI:** 10.3390/antiox14060751

**Published:** 2025-06-18

**Authors:** Uroš Glavinić, Đura Nakarada, Jevrosima Stevanović, Uroš Gašić, Marko Ristanić, Miloš Mojović, Zoran Stanimirović

**Affiliations:** 1 Department of Biology, Faculty of Veterinary Medicine, University of Belgrade, 11000 Belgrade, Serbia; uglavinic@vet.bg.ac.rs (U.G.); rocky@vet.bg.ac.rs (J.S.); mristanic@vet.bg.ac.rs (M.R.); zoran@vet.bg.ac.rs (Z.S.); 2 Center for Physical Chemistry of Biological Systems, BioScope Labs, Faculty of Physical Chemistry, University of Belgrade, 11158 Belgrade, Serbia; 3 Department of Plant Physiology, Institute for Biological Research “Siniša Stanković”, National Institute of the Republic of Serbia, University of Belgrade, 11060 Belgrade, Serbia; uros.gasic@ibiss.bg.ac.rs

**Keywords:** honey bee, grape pomace extract, EPR, antioxidant behavior, redox modulation, orbitrap

## Abstract

There is a growing interest in using agri-food by-products and a demand for natural substances that might help maintain healthy honey bee colonies. We investigated a by-product of the wine industry, a grape pomace (GP) of the autochthonous Prokupac grape cultivar from Serbia. A hydroethanolic extract (50% (*w*/*v*) ethanol) of GP (Prokupac GP extract) obtained by the pressurized liquid extraction (PLE) method was subjected to qualitative profiling of phenolic composition by liquid chromatography with OrbiTrap Exploris 120 mass spectrometer. Then, the extracts’ antioxidant and redox-modulatory activities were evaluated through Electron Paramagnetic Resonance (EPR) spectroscopy. Finally, the extract’s potential to modulate cellular redox status was evaluated using cultured AmE-711 honey bee cells. The results show that the Prokupac GP extract contains a wide array of flavonoids, anthocyanins, stilbenes, and their various conjugated derivatives and that anthocyanins, particularly malvidin-based compounds, dominate. EPR measurements showed strong scavenging activity against superoxide anion (O_2_^•−^) and hydroxyl radicals (^•^OH), with inhibition efficiencies of 84.37% and 81.81%, respectively, while activity against the DPPH radical was lower (17.75%). In the cell-based assay, the Prokupac GP extract consistently provided strong antioxidant protection and modulated the cellular response to oxidative stress by over 14%. In conclusion, while the Prokupac GP extract demonstrated antioxidant properties and the ability to modulate cellular responses to oxidative stress, in vivo studies on honey bees are required to confirm its efficacy and safety for potential use in beekeeping practice.

## 1. Introduction

At a time when the survival of pollinating insects is threatened, it is very important to help manage honey bee colonies overcome the problems they face almost all over the world. However, beekeepers have been facing significant losses of bee colonies for more than a decade, and the causes of this phenomenon are chronic and multiple [1,2]. Many causes are of such a nature that beekeepers cannot influence them, such as the shortage of natural forage (due to the decreased abundance and diversity of adequate floral resources), environmental pollution (due to industrialization, intensive agriculture, and traffic), and climatic change [1,3]. In addition, most bee colonies are affected by at least several disease agents, with the most common being viruses, endoparasite *Nosema* sp., and ectoparasite *Varroa destructor* [4,5,6], for which there are still no adequate control methods [7,8].

The mentioned stressors interact and act jointly [9,10,11,12,13,14], so the problem of bee losses cannot be solved by focusing on the individual stressor. Therefore, the only solution is to maintain honey bee colonies in good health [2] and enhance their capacity to regulate redox imbalance [15]. To achieve this, numerous honey/pollen substitutes have been tested for the capacity to compensate for the need for carbohydrates and protein, respectively [16,17,18], as well as vitamin–mineral supplements and functional feed additives that improve immunocompetence and reproductive–productive performance of honey bees or enhance their defense against parasites [19,20,21,22,23,24,25,26,27,28,29]. Although a great number of tested products were promoted as promising, there is still no consensus regarding their benefits in beekeeping practice [17,25]. The reason why these products are still not used in beekeeping practice is probably the lack of comprehensive evaluation, including the determination of their antioxidant potential. In addition, when it comes to natural-based supplements for bees, their chemical composition is usually not analyzed in detail. This study represents the first phase of testing a new product, an extract derived from the grape pomace of the autochthonous Serbian cultivar Prokupac, which we hypothesize will be beneficial as a dietary supplement for honey bees.

Grape pomace (GP), a by-product of the wine industry, is recognized as a product rich in nutritional and bioactive compounds and proposed for the development of novel health-promoting and cosmetic products considering its antioxidant, antimicrobial, antidiabetic, cardioprotective, antiproliferative, probiotic, and anti-aging potential [30,31]. However, in vivo scientific evidence for the claimed medicinal effects of GP is scarce [32]. In honey bees, only one study of the effects of GP was conducted. Encapsulated GP powder given to bees as a diet supplement appeared successful in improving their health due to the capacity to control deformed wing virus (DWV) and enhance bee immunity by upregulating the Relish gene expression [33].

The aim of this study was to comprehensively characterize the phenolic composition of Prokupac GP extract using LC-OrbiTrap MS and to evaluate its antioxidant and redox-modulatory activities through Electron Paramagnetic Resonance (EPR)-based radical scavenging assays and redox activity analysis in insect cell cultures. This integrated approach provides novel insights into the functional potential and biological impact of the extract, contributing to its prospective use in the production of dietary supplements or antioxidant therapies.

## 2. Materials and Methods

### 2.1. Chemicals and Materials

Hydrogen peroxide, absolute ethanol, 2,2-diphenyl-1-picrylhydrazyl (DPPH), 4-oxo-TEMPO (TEMPONE), and Trypan blue dye were purchased from Sigma Aldrich (Schnelldorf, Germany). 5-(Diethoxyphosphoryl)-5-methyl-1-pyrroline-N-oxide (DEPMPO) was purchased from Focus Biomolecules (Plymouth Meeting, PA, USA). Deionized water was purchased from Lonza (Verviers, Belgium). Gas-permeable Teflon capillary tubes were obtained from Zeus Industries (Orangeburg, SC, USA).

The continuous AmE-711 cell line established from honey bee (*Apis mellifera*) embryonic tissues by Goblirsch et al. [34] was provided by the Tick Cell Biobank, University of Liverpool, (Liverpool, UK). Cells were maintained in a non-humidified atmosphere at 32 °C Safegrow Pro incubator (Euroclone S.p.A., Pero, Italy) in a Schneider’s Insect Medium (Sigma-Aldrich, St. Louis, MO, USA) enriched with 10% fetal bovine serum (Sigma-Aldrich, St. Louis, MO, USA).

### 2.2. Plant Material

Prokupac grape (*Vitis vinifera* L.) pomace was obtained from a local winery located in Vlaški Do, Serbia, immediately after the wine production process. The pomace, consisting of grape skins, seeds, and stems, was collected fresh and transported to the laboratory under refrigerated conditions.

### 2.3. Sample Preparation

The fresh grape pomace was subjected to freeze-drying (lyophilization) for 48 h using a laboratory freeze dryer (Biobase, Jinan, China). The lyophilized material was then ground into a fine powder using an electric mill (Siemens, Munich, Germany). The resulting powdered pomace was stored at −80 °C prior to extraction.

### 2.4. Extraction Procedure

Extraction of bioactive compounds was performed using a pressurized liquid extraction (PLE) method. Approximately 50 g of powdered grape pomace was packed into a cotton bag and placed in the extraction chamber of the Superex F500 extractor (Superex, Karatay/Konya, Turkey). Extraction was carried out using 50% (*w*/*v*) ethanol as the solvent [35] in a solvent-to-solid ratio of 5:1 (mL/g). The extraction was conducted at a pressure of 200 bar and a temperature of 40 °C, with an initial static extraction phase lasting 30 min, followed by a dynamic phase of 60 min. After the first extraction cycle, the extract was collected, and the process was repeated using a fresh volume of solvent under the same conditions to ensure maximal recovery of active constituents. The combined extracts were subsequently freeze-dried to obtain a dry powder, corresponding to 6.4% of the initial dry weight of grape pomace. The dried extract was stored at −80 °C until further analysis.

### 2.5. LC-MS Qualitative Analysis of Phenolic Compounds in Prokupac GP Extract

Qualitative profiling of phenolic compounds in the Prokupac GP extract was carried out using a Vanquish™ Core HPLC system (Thermo Fisher Scientific, Bremen, Germany) coupled to an OrbiTrap Exoloris 120 mass spectrometer equipped with a heated electrospray ionization (HESI) probe (Thermo Fisher Scientific, Bremen, Germany). Separation was performed on a Syncronis C18 analytical column (100 × 2.1 mm, 1.7 µm particle size; Thermo Fisher Scientific, Bremen, Germany.

Chromatographic and mass spectrometric parameters were applied as previously described in the literature [36]. The tentative identification of phenolic compounds was based on monoisotopic mass and MS^2^ fragmentation patterns, with confirmation via comparison to previously reported fragmentation data [37,38,39,40]. ChemDraw software (v12.0, CambridgeSoft, Cambridge, MA, USA) was used to calculate the theoretically accurate masses, while Xcalibur software (v2.1, Thermo Fisher Scientific, Waltham, MA, USA) was used for instrument control, data acquisition, and qualitative analysis.

### 2.6. Determination of Antiradical Activity Towards DPPH Radicals

The radical scavenging efficiency of the grape pomace extract was assessed using the DPPH assay and EPR spectroscopy [35]. Briefly, 1 μL of the extract (resuspended in 50% EtOH, 1 mg/mL) was added to 29 μL of a 210 μM DPPH solution prepared in ethanol. After a 2 min incubation, the EPR signal was recorded. A control measurement was performed by adding the same volume of pure solvent instead of the extract. EPR spectra were recorded using a Bruker ELEXSYS-II E540 spectrometer (Bruker, Rheinstetten, Germany) operating in the X-band range, with the following parameters: magnetic field center at 3500 G, microwave power 10 mW, microwave frequency 9.85 GHz, modulation frequency 100 kHz, and modulation amplitude 1 G.

The antioxidant activity (AA) of the extract was calculated using the following equation:

$$AA=\frac{\left(I_{c}-I_{a}\right)}{I_{c}}\cdot 100\%$$

where $I_{c}\;$ is the double integral value of the control spectrum, and $I_{a}$ is the corresponding value for the extract-treated sample, both derived from the EPR signal.

### 2.7. Determination of Antiradical Activity Towards Hydroxyl Radicals

To evaluate the capacity of Prokupac GP extract to remove ^•^OH radicals, a UV–Vis generator system containing the spin trap DEPMPO was deployed, adopting a procedure similar to the one available in the literature [41,42]. The control EPR signal for hydroxyl radicals was generated by mixing 1 μL of the spin trap DEPMPO, 27 μL of distilled water, and 2 μL of a diluted hydrogen peroxide (H_2_O_2_) solution (2.2 μL/mL), followed by UV irradiation to induce radical formation. The prepared mixture was exposed to UV light for 30 s, and the EPR signal was recorded after 5 min.

To evaluate the scavenging activity of the grape pomace extract, the same procedure was repeated with the substitution of 1 μL of extract in place of water. The EPR measurements were performed under identical conditions as described for the DPPH radical assay.

### 2.8. Determination of Antiradical Activity Towards Superoxide Anion Radicals

To evaluate the scavenging activity against superoxide anion radicals (O_2_^•−^) [41], 1 μL of the grape pomace extract was added to 29 μL of a reaction mixture containing 18 μL of distilled water, 10 μL of concentrated hydrogen peroxide (H_2_O_2_), and 1 μL of the spin trap DEPMPO. The mixture was then exposed to UV irradiation for 30 s to induce radical formation.

After irradiation, the reaction mixture was transferred into a gas-permeable Teflon capillary tube, and the EPR spectrum was recorded 2 min later. A control spectrum was recorded in the same manner, replacing the extract with an equal volume of water. EPR measurements were conducted under the same conditions as those described for the DPPH radical assay.

### 2.9. Measurement of Cell Viability

The trypan blue exclusion test [43] was utilized to determine the cytotoxicity of Prokupac GP extract towards AmE-711 honey bee cells. Briefly, 25 μL of the cell suspension (1 × 10^6^ cells/mL) was treated with an equal volume of Prokupac GP extract at a concentration of 10 mg/mL. Cells in the negative control were mixed with PBS only, while cells in the positive control were treated with 100 μM hydrogen peroxide. The cells were incubated for 1 h at 32 °C. Consequently, 250 μL of 0.4% Trypan blue dye was mixed with 25 μL of cell suspension and 725 μL of PBS. After a 5 min incubation, the number of dead (stained) and viable (non-stained) cells was counted in a Neubauer chamber. The measurements were repeated three times.

### 2.10. Redox Response of Apis mellifera Cells to Prokupac GP Extract Treatment

The redox activity of *Apis mellifera* cells (AmE-711) was investigated using EPR spectroscopy. Cells were cultured in Schneider’s Insect Medium and adjusted to a final concentration of 100,000 cells in 27 µL of medium. All experimental samples had a total volume of 30 µL. The spin probe TEMPONE was used at a final concentration of 0.025 mM, added as 1 µL of a 0.75 mM solution [44]. An aqueous extract of *Vitis vinifera* cv. Prokupac grape pomace was prepared by dissolving 10 mg of dry extract powder in 1 mL of deionized water and used to evaluate its redox-modulating activity. Hydrogen peroxide was used to induce oxidative stress, prepared by diluting 1 µL of 10 M H_2_O_2_ in 500 µL of deionized water. All measurements were performed using sterile, deionized water.

Samples were prepared as follows to assess redox activity, oxidative stress, and the modulating effects of the grape extract. All samples were recorded two minutes after adding TEMPONE.

- Controls:
  ○ Water Control: 29 µL deionized water + 1 µL TEMPONE.
  ○ Medium Control: 27 µL Schneider’s Insect Medium + 2 µL deionized water + 1 µL TEMPONE.
  ○ Cell Baseline: 27 µL cell suspension (100,000 cells) + 2 µL deionized water + 1 µL TEMPONE.
- Grape Extract Effects:
  ○ Extract in Water: 28 µL deionized water + 1 µL grape extract + 1 µL TEMPONE.
  ○ Extract in Medium: 27 µL Schneider’s Insect Medium + 1 µL grape extract + 1 µL deionized water + 1 µL TEMPONE.
  ○ Extract with Cells: 27 µL cell suspension + 1 µL grape extract + 1 µL deionized water + 1 µL TEMPONE.
- Cells Under Oxidative Stress:
  ○ Cell + H_2_O_2_: 27 µL cell suspension + 1 µL diluted H_2_O_2_ + 1 µL deionized water + 1 µL TEMPONE.
  ○ Cell + Extract + H_2_O_2_: 27 µL cell suspension + 1 µL grape extract + 1 µL diluted H_2_O_2_ + 1 µL TEMPONE.

EPR measurements were conducted using a Bruker ELEXSYS II X-band spectrometer operating at a microwave frequency of 9.8 GHz. Acquisition parameters were as follows: microwave power 10 mW, modulation frequency 100 kHz, and modulation amplitude 2 G.

### 2.11. Statistical Analysis

All experiments were repeated three times, and the values presented here are the means of the three independent measurements. The results are expressed as means with standard error (±SE). Statistical significance was analyzed using Statistica 12.0 64-bit Statistical Software (StatSoft (Europe) GmbH, Hamburg, Germany).

## 3. Results and Discussion

### 3.1. Chemical Profiling of Prokupac GP Extract

The qualitative UHPLC-LTQ Orbitrap MS analysis of Prokupac grape pomace (GP) extract (Table 1) revealed a diverse profile of phenolic compounds, encompassing flavonoid glycosides and aglycones, anthocyanins and pyranoanthocyanins, stilbenes, and their various glycosylated and acylated derivatives. A total of 41 metabolites were identified, and Table 1 shows the relative abundance of these metabolites in the examined sample. This rich diversity is consistent with previously reported phenolic profiles in red grape pomace and underscores its potential as a valuable source of bioactive compounds for nutraceutical and cosmetic applications [31].

The phenolic profile of the Prokupac grape pomace (GP) extract is dominated by anthocyanins, accounting for over 95% of all identified compounds. Among them, malvidin 3-*O*-glucoside stands out as the most abundant individual constituent (37.93%), followed by malvidin 3-*O*-(6″-*O*-p-coumaroyl)-glucoside (22.21%), malvidin 3-*O*-(6″-*O*-acetyl)-glucoside (12.24%), peonidin 3-*O*-glucoside (5.94%), peonidin 3-*O*-(6”-*O*-*p*-coumaroyl)-glucoside (4.41%), delphinidin 3-*O*-glucoside (3.34%), petunidin 3-*O*-glucoside (2.77%), and malvidin acetylglucoside (2.39%). These anthocyanins are well-recognized for their antioxidant, anti-inflammatory, and cardioprotective effects [45,46,47]. Malvidin 3-*O*-glucoside, in particular, is known for its strong free radical scavenging activity and ability to stabilize reactive oxygen species due to its hydroxylated aromatic structure [47]. It has been already detected as the most abundant anthocyanin compound in grape varieties cultivated in the Balkan region [46]. The presence of these compounds contributes to the intense coloration and polyphenolic density characteristic of Prokupac wines. Malvidin derivatives have strong electron-donating groups (methoxy and hydroxyl), contributing to radical scavenging [48]. The diversity of anthocyanins, including complex derivatives like malvidin 3-*O*-(6″-*O*-acetyl)-glucoside and petunidin 3-*O*-(6″-*O*-*p*-coumaroyl)-glucoside, highlights their structural variety, which may enhance pigment stability and provide additional functional benefits, such as photoprotection and antimicrobial properties [45,46]. A high correlation between total anthocyanin content and the DPPH· scavenging ability of red wines has already been confirmed [46].

In addition to anthocyanins, the extract contains notable quantities of flavonols such as quercetin (0.16%) and its glycosides (quercetin-3-*O*-galactoside, 0.15%; quercetin-3-*O*-glucoside, 0.09%). Quercetin is one of the most studied dietary flavonoids, valued for its potent antioxidant, anti-carcinogenic, antiviral, neuroprotective, pulmonary, and cardiovascular benefits [49,50]. In particular, quercetin’s capacity to neutralize highly reactive species like peroxynitrite and hydroxyl radicals is believed to contribute to these potential positive health effects [50]. Although present in lower concentrations, the presence of aglycone quercetin is particularly relevant due to its higher bioactivity compared to its glycosylated forms [51].

Luteolin (1.19%), a flavone detected in significant amounts, also contributes to the extract’s bioactivity. It is known for its anti-inflammatory, antioxidant, anti-allergy, and anti-cancer properties, and its relatively simple structure with multiple hydroxyl groups enhances its radical scavenging potential [52]. Direct evidence showing luteolin as a very potent ROS scavenger and lipid peroxidation inhibitor has been demonstrated both in vivo and ex vivo, with a significant correlation [53]. Other compounds such as catechin (0.53%) and gallic acid (0.11%) are also of interest. Catechin is a well-known flavanol with strong antioxidant and metal-chelating activities [54], while gallic acid is a potent phenolic acid with anti-inflammatory and antimicrobial effects [55]. The extract also includes phenolic acids like syringic acid (0.29%) and vanillic acid (0.14%), which are known for their moderate antioxidant activity and potential synergistic interactions with other phenolics. Syringic acid exhibits antimitogenic activity against human breast, colorectal, and malignant melanoma cells and has significant chemopreventive effects in carcinogenesis [56]. The antioxidant activity of syringic acid has been shown to hamper the oxidative stress markers in L-arginine–induced acute toxicity [57]. The antioxidant and anti-inflammatory potential of vanillic acid was explored in vitro and ex vivo in human immune cells and non-cellular models [58]. These findings indicate a synergic role of vanillic acid in protection against chronic diseases related to oxidative stress and inflammation.

Overall, the qualitative analysis demonstrates that Prokupac GP is a rich source of structurally diverse phenolic compounds, with anthocyanins being a particularly prominent class. The dominance of malvidin derivatives, combined with the presence of flavonols, flavones, flavanols, and phenolic acids, suggests a high potential for antioxidant activity and broader biological efficacy. The presence of these phenolics supports the potential for value-added applications of this winemaking by-product. These findings encourage further exploration of targeted extraction and formulation strategies to harness Prokupac GP-derived phenolics for functional foods, dietary supplements, or cosmetic products.

### 3.2. Antiradical Activity of Prokupac GP Extract

**Figure 1:**
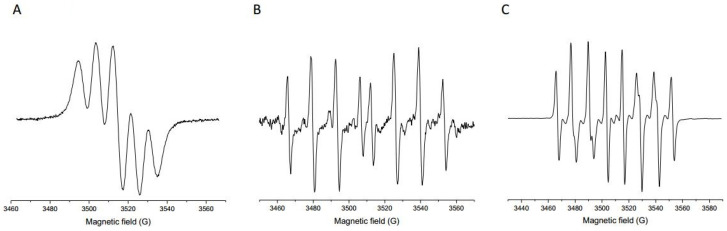
Representative EPR spectra of selected radical species and/or their spin-adducts: DPPH (**A**), DEPMPO/OH (**B**), and DEPMPO/OOH (**C**).

**Figure 2:**
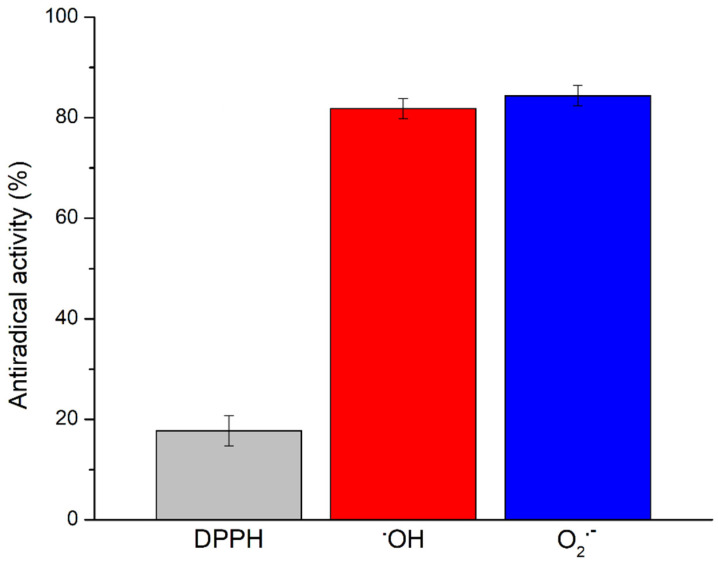
Antiradical activity (in %) of Prokupac GP extract towards selected radical species. Data are presented as mean ± SE (*n* = 3 independent experiments).

The antioxidant potential of the Prokupac GP extract was evaluated by measuring their radical scavenging activities against three major reactive species: DPPH, hydroxyl radicals (^•^OH), and superoxide anion radicals (O_2_^•−^), using EPR spectroscopy. The representative EPR spectra of studied radical species and/or their spin-adducts are shown in Figure 1, while the percentage of inhibition observed for each radical type is summarized in Figure 2.

Among the tested radical species, the Prokupac GP extract exhibited the highest scavenging activity against superoxide anion radicals (O_2_^•−^), with an inhibition efficiency of 84.37%, closely followed by hydroxyl radicals (^•^OH) at 81.81%. In contrast, its activity against the DPPH radical was markedly lower, with only 17.75% inhibition observed. This pattern suggests that the extract contains antioxidant compounds with higher specificity or reactivity towards oxygen-centered radicals such as ^•^OH and O_2_^•−^, which are more biologically relevant and reactive under physiological oxidative stress conditions [59,60]. The relatively low activity against the DPPH radical likely reflects both the radical’s different chemical nature and the limitations of the DPPH assay in evaluating hydrophilic or complex polyphenolic matrices, particularly in aqueous systems [61,62]. These findings highlight the importance of using multiple radical assays to characterize antioxidant activity comprehensively, as individual probes vary in chemical properties and relevance to biological oxidative stress [61,63].

Overall, the findings highlight the Prokupac GP extract as a potent scavenger of reactive oxygen species, particularly hydroxyl and superoxide radicals. These results underscore the potential of grape pomace as an industrial by-product as a valuable source of natural antioxidants for pharmaceutical, nutraceutical, or cosmetic applications.

EPR spectroscopy has not been usually deployed in screening the antioxidant activity of products tested for supplementation of honey bee feed. In fact, only one of them has been analyzed by EPR spectroscopy so far, the extract of *Agaricus bisporus* [64], demonstrating significant antiradical activity toward DPPH (44.49%) and ^•^OH (59.39%). Compared to *A. bisporus* extract, the Prokupac GP extract scavenged less DPPH (17.75%) and significantly more ^•^OH (81.81%), indicating its higher selectivity toward ^•^OH, which is a more biologically relevant radical species.

### 3.3. Cell Viability Measurement Results

The viability of AmE-711 cells in the negative control and the group treated with Prokupac GP extract for one hour prior to staining with Trypan blue exclusion dye was >90%, while in the positive control (treated with 100 μM H_2_O_2_), it was above 85%. Therefore, in all experimental groups, the level of cytotoxicity was acceptable and proved that the design is appropriate for further analyses in this study.

### 3.4. Redox Modulation in Apis Mellifera Cells by Prokupac GP Extract

**Figure 3:**
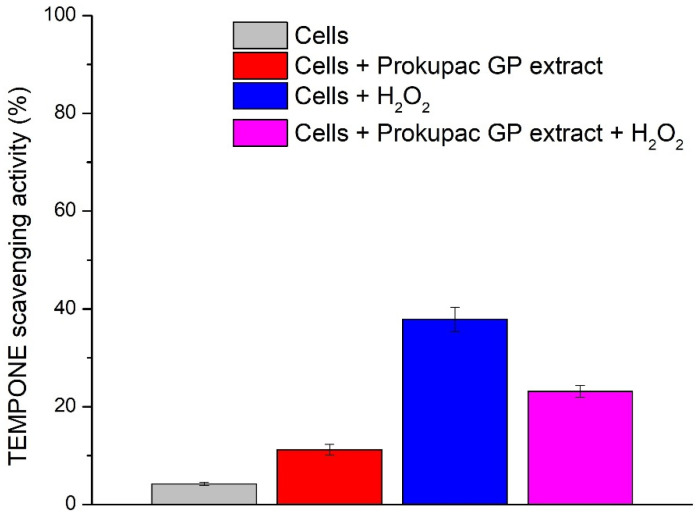
Effect of Prokupac GP extract on TEMPONE oxidation in AmE-711 honey bee cells under basal and oxidative stress conditions. Data are presented as mean ± SE (*n* = 3 independent experiments). “Cells” refers to untreated control cells; “Cells + Prokupac GP extract” refers to cells treated with Prokupac grape pomace extract; “Cells + H_2_O_2_” refers to cells exposed to hydrogen peroxide to induce oxidative stress; and “Cells + Prokupac GP extract + H_2_O_2_” refers to cells pretreated with Prokupac extract followed by hydrogen peroxide exposure.

The redox activity of AmE-711 honey bee cells and the modulatory effects of Prokupac GP extract were evaluated using EPR spectroscopy and the spin probe TEMPONE (Figure 3). This approach has been deployed for the first time and enables direct and sensitive detection of oxidative changes in biological systems and is well-suited for studying cell redox status and antioxidant interactions.

In live cell suspensions, TEMPONE signal reduction was lowest (4.20%), consistent with a well-maintained intracellular redox balance. The addition of the Prokupac GP extract led to a greater reduction in TEMPONE signal (11.19%), likely reflecting mild antioxidative properties of Prokupac and/or redox modulation. This shift may result from extract-induced metabolic responses or direct redox interactions with cellular components. Under oxidative stress conditions (cells + H_2_O_2_), the TEMPONE signal was markedly reduced (37.88%), confirming a peroxide-induced stress response. Co-treatment with the Prokupac GP extract mitigated this effect by 14.79%, resulting in a lesser signal reduction (23.09%) and demonstrating a protective antioxidant effect. The superior performance of the Prokupac GP extract is likely due to its high content of anthocyanins, flavonoids, and stilbenes—compounds known for their potent ROS-scavenging abilities [40,65,66]. Control experiments involving culture medium and medium with peroxide (without cells) showed no significant changes in the TEMPONE signal.

Overall, these findings highlight the redox sensitivity of *Apis mellifera* cells, the oxidative impact of hydrogen peroxide, and the context-dependent antioxidant effects of the tested natural-based extract. Notably, the Prokupac GP extract provided strong antioxidant protection, underscoring its potential utility in mitigating oxidative stress, usually induced in honey bees by agropesticides [67,68] and parasites, especially *Nosema* sp. microsporidians [20,22,69]. There are other natural (plant-based) products reported to be efficient in mitigating oxidative stress in honey bees and activating their antioxidant system in laboratory cage and field experiments [20,22,23,69,70,71], but their effect was not tested with such a high-resolution method as in the case for Prokupac GP extract in this study. However, the AmE-711 cell line is derived from honey bee embryos, so it does not fully reflect adult bee cells. This limitation can be addressed by testing the effects of Prokupac GP extract on live bees both in laboratory conditions (on caged bees) and in the field (on full-sized bee colonies). Only after such testing can its antioxidant effectiveness be compared with that of other natural-based supplements, and its potential use in beekeeping can be considered.

## 4. Conclusions

This study highlights the strong antioxidant potential of Prokupac grape pomace (GP) extract, which is rich in diverse phenolic compounds, particularly anthocyanins and flavonoids. LC-OrbiTrap MS analysis confirmed a broad range of bioactives, supporting its relevance for nutraceutical, pharmaceutical, and cosmetic applications. The extract showed pronounced scavenging activity against superoxide anion and hydroxyl radicals, while its weaker performance against the DPPH radical emphasizes the importance of using multiple radical models to evaluate antioxidant properties.

The cell-based redox activity assay further reinforced these findings. In honey bee cell cultures, the Prokupac GP extract consistently modulated cellular redox activity and protected cells from excessive activation of redox defense mechanisms under hydrogen peroxide-induced oxidative stress. This protective effect is likely driven by the extract’s rich anthocyanin and flavonoid content. These findings establish Prokupac GP extract as a promising natural antioxidant for both acellular and cellular systems.

Future research should include further optimization of the extraction process, particularly the solvent mixture composition, to enhance the yield and selectivity of bioactive compounds. Additionally, in vivo studies in honey bees are warranted to explore the extract’s potential to promote oxidative stress resilience and overall colony health.

## Figures and Tables

**Table 1:** LC-MS data on metabolites identified in Prokupac grape pomace extract.

No	Compound Name	*t*_R_, min	Molecular Formula, [M ± H]^±^	Calculated Mass, *m*/*z*	Exact Mass, *m*/*z*	Δ mDa	MS^2^ Fragments, (% Base Peak)	Relative Content, %
Flavonoid glycosides								
1	Myricetin 3-*O*-glucuronide	6.69	C21H19O14+	495.07696	495.07114	5.82	319.04129 (100)	0.06
2	Myricetin 3-*O*-glucoside	6.70	C21H21O13+	481.09779	481.09215	5.64	153.01657 (8), 217.04716 (3), 245.04181 (4), 273.03644 (3), 319.04123 (100)	0.64
3	Myricetin 3,4′-di-*O*-glucoside	6.71	C27H31O18+	643.15052	643.14488	5.64	153.01663 (3), 319.04135 (100), 481.09253 (46)	0.10
4	Laricitrin 3-*O*-glucoside	6.96	C22H23O13+	495.11334	495.10778	5.57	245.04189 (3), 301.03110 (4), 318.03412 (7), 333.05667 (100)	0.67
5	Kaempferol 3-*O*-glucoside	7.01	C21H21O11+	449.10787	449.10273	5.13	153.01622 (4), 287.05197 (100)	0.06
6	Syringetin 3-*O*-(6”-*O*-acetyl)-glucoside	7.48	C25H27O14+	551.13956	551.13314	6.42	347.07251 (100)	0.08
Flavonoid aglycones								
7	Epicatechin	6.40	C15H15O6+	291.08634	291.08292	3.42	91.05327 (10), 119.04826 (11), 123.04277 (79), 139.03754 (100), 147.04240 (18), 165.05298 (11)	0.11
8	Myricetin	6.71	C15H11O8+	319.04487	319.04112	3.75	153.01646 (23), 217.0462 (10), 245.04092 (5), 273.03641 (4), 319.04129 (100)	0.41
9	Eriodictyol	7.34	C15H13O6+	289.07079	289.06748	3.31	107.0479 (95), 149.02167 (70), 153.01648 (100), 215.06883 (46), 217.12192 (14), 243.06279 (50)	0.05
10	Luteolin	7.55	C15H11O6+	287.05504	287.05180	3.24	123.00639 (18), 139.05305 (7), 183.02797 (7), 287.05179 (100)	1.19
11	Quercetin	7.83	C15H11O7+	303.04996	303.04658	3.38	137.02168 (8), 153.01657 (15), 229.04723 (9), 257.04175 (3), 303.04657 (100)	0.16
12	Kaempferol	8.21	C15H11O6+	287.05504	287.05181	3.24	121.02708 (11), 137.02194 (3), 153.01660 (16), 287.05185 (100)	0.10
13	Isorhametin	8.28	C16H13O7+	317.06561	317.06203	3.58	153.01654 (25), 217.04721 (7), 229.04698 (7), 245.04184 (6), 302.03873 (8), 317.06192 (100)	0.07
14	Daidzein	9.04	C15H11O4+	255.06519	255.06232	2.86	129.03270 (9), 153.01711 (22), 255.06216 (100)	0.03
Anthocyanins and pyranoanthocyanins								
16	Delphinidin 3-*O*-glucoside	5.98	C21H21O12+	465.10288	465.09737	5.51	229.04707 (4), 303.04663 (100), 465.09833 (7)	3.34
17	Cyanidin 3-*O*-glucoside	6.18	C21H21O11+	449.10787	449.10260	5.27	287.05188 (100)	0.67
18	Petunidin 3-*O*-glucoside	6.25	C22H23O12+	479.11843	479.11283	5.60	245.04167 (4), 274.04401 (7), 302.03854 (16), 317.06171 (100), 479.11267 (8)	2.77
19	Peonidin 3-*O*-glucoside	6.43	C22H23O11+	463.12352	463.11824	5.27	258.04932 (8), 286.04398 (19), 301.06711 (100), 463.11801 (9)	5.94
20	Malvidin 3-*O*-glucoside	6.44	C23H25O12+	493.13418	493.12901	5.17	242.05501 (5), 270.04877 (4), 287.05194 (6), 315.04642 (12), 331.07739 (100), 493.12900 (11)	37.93
21	Delphinidin 3-*O*-(6″-*O*-acetyl)-glucoside	6.57	C23H23O13+	507.11334	507.10763	5.72	257.04181 (3), 303.04630 (100), 507.1066 (19)	0.16
22	Delphinidin 3,5-di-*O*-glucoside	6.63	C27H31O17+	627.15560	627.14923	6.37	303.04630 (100), 465.09827 (41), 627.15192 (19)	0.06
23	Malvidin 3-*O*-glucoside-pyruvic acid (Vitisin A)	6.65	C26H25O14+	561.12391	561.11712	6.79	383.03549 (11), 399.06644 (100), 561.11633 (8)	0.11
24	Malvidin 3-*O*-glucoside-acetaldehyde (Vitisin B)	6.69	C25H25O12+	517.13418	517.12800	6.18	266.05453 (7), 294.04871 (3), 311.05157 (4), 339.04639 (20), 355.07745 (100), 517.12842 (12)	0.05
25	Cyanidin 3-*O*-(6″-*O*-acetyl)-glucoside	6.72	C23H23O12+	491.11843	491.11284	5.59	287.05200 (100)	0.10
26	Petunidin 3-*O*-(6″-*O*-acetyl)-glucoside	6.75	C24H25O13+	521.12909	521.12297	6.12	274.04404 (6), 302.03873 (20), 317.06186 (100), 521.12262 (26)	0.62
27	Malvidin 3-*O*-(6″-*O*-acetyl)-glucoside-pyruvic acid	6.80	C28H27O15+	603.13447	603.12963	4.84	310.04498 (5), 338.03796 (3), 355.04129 (4), 383.03543 (11), 399.06668 (100), 603.12836 (21)	0.03
28	Malvidin 3-*O*-(6″-*O*-acetyl)-glucoside	6.90	C25H27O13+	535.14464	535.13868	5.97	242.05484 (4), 270.04877 (4), 287.05176 (6), 315.04575 (14), 331.07773 (100), 535.13867 (31)	12.24
29	Peonidin 3-*O*-(6″-*O*-acetyl)-glucoside	6.90	C24H25O12+	505.13418	505.12832	5.86	286.04416 (22), 301.06726 (100), 505.12970 (23)	1.99
30	Delphinidin 3-*O*-(6″-*O*-*p*-coumaroyl)-glucoside	6.99	C30H27O14+	611.13956	611.13474	4.82	303.04639 (100), 611.13330 (46)	0.29
31	Malvidin 3-*O*-(6″-*O*-caffeoyl)-glucoside	7.06	C32H31O15+	655.16579	655.15982	5.96	287.05182 (3), 315.04565 (4), 331.07724 (100), 655.15771 (47)	0.70
32	Cyanidin 3-*O*-(6″-*O*-*p*-coumaroyl)-glucoside	7.11	C30H27O13+	595.14464	595.13782	6.82	287.05185 (100), 595.13727 (31)	0.23
33	Petunidin 3-*O*-(6″-*O*-*p*-coumaroyl)-glucoside	7.13	C31H29O14+	625.15521	625.14977	5.43	302.03879 (12), 317.06210 (100), 625.14832 (41)	1.02
34	Malvidin 3-*O*-(6″-*O*-*p*-coumaroyl)-glucoside-pyruvic acid	7.14	C35H31O16+	707.16078	707.15654	4.24	399.06665 (100), 707.15253 (39)	0.07
35	Malvidin 3-*O*-(6″-*O*-feruloyl)-glucoside	7.23	C33H33O15+	669.18142	669.17678	4.64	287.05356 (3), 316.05267 (4), 331.07776 (100), 669.17291 (46)	0.09
36	Malvidin 3-*O*-(6″-*O*-*p*-coumaroyl)-glucoside	7.27	C32H31O14+	639.17086	639.16558	5.28	315.04639 (4), 331.07748 (100), 639.16357 (42)	22.21
37	Peonidin 3-*O*-(6″-*O*-*p*-coumaroyl)-glucoside	7.28	C31H29O13+	609.16039	609.15525	5.14	286.04407 (14), 301.06726 (100), 609.15338 (43)	4.41
Stilbenes								
38	Piceatanol	6.41	C14H11O4-	243.06630	243.06328	3.02	175.07425 (6), 199.07414 (5), 201.05354 (13), 243.06316 (100)	0.12
39	Resveratrol 3-*O*-glucoside (Polydatin)	6.58	C20H21O8-	389.12420	389.11963	4.57	227.06857 (100)	0.39
40	Resveratrol	7.28	C14H11O3-	227.07140	227.06869	2.71	143.04865 (6), 159.07988 (3), 183.07947 (6), 185.05856 (21), 227.06863 (100)	0.10
41	Viniferin	7.48	C28H21O6-	453.13440	453.12864	5.76	359.08707 (18), 369.10822 (19), 385.13977 (18), 411.11874 (40), 435.11868 (29), 453.12857 (100)	0.67

## Data Availability

All data underlying the results are available as part of the article and no additional source data are required.
